# Adverse reactions of four multi-targeted tyrosine kinase inhibitors: a descriptive analysis of the WHO-VigiAccess database

**DOI:** 10.3389/fphar.2025.1585862

**Published:** 2025-04-22

**Authors:** Lijun Li, Jiayu Bai, Xuelong Wen, Xuefan Zeng

**Affiliations:** ^1^ Department of Pharmacy, The Second Affiliated Hospital, University of South China, Hengyang, Hunan, China; ^2^ Hengyang Medical School, University of South China, Hengyang, Hunan, China; ^3^ Hunan Provincial Key Clinical Laboratory of Basic and Clinical Pharmacological Research of Gastrointestinal Cancer, the Second Affiliated Hospital, University of South China, Hengyang, Hunan, China; ^4^ Department of rehabilitation medicine, The First Affiliated Hospital of Dalian Medical University, Dalian, Liaoning, China; ^5^ Wuxi School of Medicine, Jiangnan University, Wuxi, Jiangsu, China; ^6^ Chongqing Medical University, Chongqing, China

**Keywords:** MTKIs, renal cell carcinoma, adverse drug reactions, WHO-Vigiaccess, ROR

## Abstract

**Background:**

The introduction of multi-targeted tyrosine kinase inhibitors (MTKIs) such as axitinib, lenvatinib, sorafenib, and sunitinib has greatly broadened the available treatment options for Renal Cell Carcinoma (RCC). The study aims to compare the nature of the adverse reactions associated with these four MTKIs to identify which medication poses the least risk for personalized patient management, thus enabling more accurate clinical drug oversight.

**Methods:**

Employing a retrospective descriptive analysis methodology, this research concentrated on four commercially available MTKIs. Reports pertaining to these medications were sourced from the WHO-VigiAccess database. The data gathering process involved collecting comprehensive information on various parameters, such as age demographics, gender, and the geographical distribution of patients associated with the ADR reports. Furthermore, the study explored disease systems and symptoms that were documented alongside the adverse reactions, as outlined in the annual ADR reports produced by the WHO. To assess the relationship between these four MTKIs and the linked AEs, both the Proportional Reporting Ratio (PRR) and the Reported Odds Ratio (ROR) were utilized.

**Results:**

At the time of the search, a total of 123,818 AEs associated with the four MTKIs had been documented in the VigiAccess database. The common ADRs for these four MTKIs include diarrhoea, fatigue, death, hypertension, nausea, asthenia, weight decreased, and vomiting. Gastrointestinal disorders and general disorders and administration site conditions emerged as the SOCs with the highest number of adverse signals, both ranking first in terms of frequency. The elevated ROR (1.08) and PRR (1.06) values associated with gastrointestinal disorders in patients treated with sorafenib suggest a higher incidence of such adverse events compared to those observed with axitinib, lenvatinib, and sunitinib.

**Conclusion:**

Recent comparative observational research suggests that the ADR reports submitted to the WHO and the FDA for these medications highlight both common and specific ADRs. It is essential for clinical practitioners to develop personalized treatment strategies that consider the adverse effects linked to different medications, alongside the unique circumstances of their patients, thus encouraging the responsible use of these MTKIs.

## Introduction

Renal Cell Carcinoma (RCC) stands out as the most prevalent malignant tumor affecting the kidneys, representing over 90% of all renal cancers. This type of cancer arises from the epithelial cells found within the renal tubules and showcases considerable heterogeneity, alongside a notable resistance to standard chemotherapy and radiotherapy treatments (Hsieh et al., 2017). The underlying mechanisms of RCC are complex, with the overproduction of Vascular Endothelial Growth Factor (VEGF) and Vascular Endothelial Growth Factor Receptor (VEGFR) playing a pivotal role in the processes of tumor angiogenesis and development. Remarkably, around 25% of patients diagnosed with RCC present with either locally advanced or metastatic disease, and between 20% and 40% of individuals with localized primary tumors will ultimately experience the spread of metastases. Given the frequently asymptomatic progression and the unfavorable outlook linked to advanced or metastatic RCC, the range of treatment options remains quite limited. Among the various subtypes of RCC, Clear Cell Renal Cell Carcinoma (ccRCC) is the most common, representing approximately 70%–80% of all RCC instances. ccRCC is distinguished by significant vascularization, and its development is closely linked to hereditary von Hippel-Lindau (VHL) disease. In ccRCC specifically, the loss of function of the VHL tumor suppressor gene leads to the excessive buildup of Hypoxia-Inducible Factor (HIF) (Sato et al., 2013). Under normal physiological circumstances, HIF serves as a transcription factor that enhances the expression of multiple pro-angiogenic factors, such as VEGF and Platelet-Derived Growth Factor (PDGF) (Chen et al., 2023). As a result, in most ccRCC cases, dysregulation of signaling pathways caused by mutations in the VHL gene facilitates angiogenesis, as well as the survival, proliferation, and differentiation of cancerous cells. The introduction of Multi-Targeted Tyrosine Kinase Inhibitors (MTKIs) has brought about a significant change in the treatment approach for RCC. MTKIs function by targeting an array of protein kinases, such as the VEGFR, PDGFR, and the Stem Cell Factor Receptor (c-KIT) (Faivre et al., 2007). Presently, commonly utilized MTKIs include axitinib, lenvatinib, sorafenib, and sunitinib, all of which have demonstrated considerable clinical effectiveness in the management of advanced or metastatic RCC. By inhibiting the activities of VEGFR, PDGFR, and other associated kinases, these agents promote their antitumor effects, hindering tumor angiogenesis and growth while triggering apoptosis (Bahadoram et al., 2022). As a result, they offer novel therapeutic avenues for patients facing advanced RCC. Despite the thoroughness of pre-marketing clinical trials, the safety of these medications remains partially undefined based on data from pre-authorization studies, as these trials are performed in controlled conditions that differ from everyday practice (Gagliardi et al., 2022). Four MTKIs have been available on the market for a considerable period, catering to a wide range of patients and serving various purposes. Consequently, it is crucial and informative to perform safety research by utilizing extensive real-world data. Therefore, a more thorough characterization of the ADRs associated with these MTKIs is necessary, utilizing spontaneous reports gathered from pharmacovigilance databases. It is important to highlight that comparative studies addressing the similarities and differences in ADRs induced by these medications are notably lacking. Since 2015, the data stored in VigiBase has been accessible to the public through VigiAccess (Watson et al., 2018; Habarugira and Figueras, 2021). The VigiAccess database facilitates searches using the trade names of drugs, while also identifying the active ingredients and presenting the corresponding results of ADR reports. This research primarily focuses on four MTKIs: axitinib, lenvatinib, sorafenib, and sunitinib. Clinicians often need to tailor treatment options based on the potential risk of AEs for each patient. To assess the occurrence of adverse reactions associated with these MTKIs, we conducted a descriptive study that analyzed spontaneously reported adverse reactions in the VigiAccess database and compared the rates of adverse reactions linked to these four MTKIs. Furthermore, we employed the Proportional Reporting Ratio (PRR) and the Reported Odds Ratio (ROR) to evaluate the relationship between these four MTKIs and the associated AEs.

## Materials and methods

### Drug sample

Table 1 presents the four MTKIs that we have studied and are currently available for clinical use. Axitinib is a potent and selective second-generation VEGFR inhibitor that targets VEGFR-1, VEGFR-2, and VEGFR-3, while exhibiting a weaker inhibitory effect on PDGFR. It is primarily utilized as a second-line treatment for advanced RCC (Schmidt et al., 2018). In the AXIS Phase III clinical trial, axitinib significantly extended the progression-free survival (PFS) of patients, achieving a median PFS of 6.7 months. Lenvatinib targets multiple receptor tyrosine kinases, including VEGFR, FGFR, and PDGFR, thereby inhibiting tumor angiogenesis and cell proliferation by blocking these targets. It is widely employed in the treatment of RCC, thyroid cancer, and hepatocellular carcinoma (Romero, 2019). In 2015, lenvatinib received FDA approval for the second-line treatment of advanced RCC. Based on Phase II clinical trial data, the median PFS for the lenvatinib combination therapy group reached 14.6 months, significantly surpassing that of the monotherapy group (Motzer et al., 2015). Sorafenib is the first MTKI approved for advanced RCC, targeting VEGFR, PDGFR, and RAF kinase. In 2005, sorafenib received FDA approval based on the results of the Phase III TARGET trial, which demonstrated that the median PFS for the sorafenib group was 5.5 months, significantly longer than the 2.8 months observed in the placebo group, with a disease control rate of 84%. Although subsequent drugs have surpassed sorafenib in certain metrics, it remains a crucial option for the treatment of advanced renal cancer (Wilhelm et al., 2006). Sunitinib is an oral MTKI primarily targeting VEGFR, PDGFR, KIT, and FLT3. In 2006, sunitinib received FDA approval for the first-line treatment of advanced RCC. Key Phase III trials indicated that the median PFS for the sunitinib group was 11 months, significantly better than the 5 months reported for the interferon-α group. Due to its inhibitory effect on KIT, sunitinib is also approved for the second-line treatment of gastrointestinal stromal tumors (Moran et al., 2019).

**TABLE 1:** General information of four MTKIs.

Active ingredients	Brand names	Chemical formula	Drug targets	Main conditions	The earliest year on the market
Axitinib	Inlyta, Axinix	C_22_H_18_N_4_OS	VEGFR and PDGFR	Renal cell carcinoma, Pancreatic cancer	2012
Lenvatinib	Lenvima	C_22_H_23_ClN_4_O_7_S	VEGFR, FGFR, PDGFR, RET	Thyroid cancer, Renal cell carcinoma, Hepatocellular carcinoma	2015
Sorafenib	Nexavar	C_28_H_24_ClF_3_N_4_O_6_S	VEGFR, PDGFR, RAF	Renal cell carcinoma, Hepatocellular carcinoma, Thyroid cancer	2005
Sunitinib	Sutent	C_26_H_33_FN_4_O_7_	VEGFR, PDGFR, KIT and FLT3	Renal cell carcinoma, Gastrointestinal stromal tumor, Pancreatic cancer	2006

### Data sources

The WHO-VigiAccess database was searched on 17 February 2025, to gather all documented AEs that occurred following the introduction of four MTKIs. The access URL is https://www.vigiaccess.org. All pharmaceutical agents under study were identified using their generic names. Data collection spanned different age ranges, genders, years of reporting, and geographic regions, as detailed by WHO-VigiAccess. Descriptive statistics were computed utilizing Excel 2021.

WHO-VigiAccess serves as an open-access portal to the PIDM database, facilitating the retrieval of safety reports concerning medicinal products provided by the UMC. The evaluation was based on system organ class (SOC) and preferred terms (PTs) as defined by the Medical Dictionary for Regulatory Activities (MedDRA). As a result, records for each MTKI were gathered, and all distinct AEs noted at the MedDRA SOC and PT levels were pinpointed to outline the range of toxicities. The reporting terms employed in MedDRA were compiled from various dictionaries, including the WHO Adverse Reaction Terminology (WHO-ART) and others (Sultana et al., 2020). In total, 27 items were classified by SOC. This research concentrated on the PTs, which represent the extent of publicly available information in the VigiBase database via WHO-VigiAccess. To assess the outcomes of the identified safety signals, we organized them using outcome codes, culminating in three essential categories: death, hospitalization, and major events, which encompass life-threatening occurrences, disabilities, and congenital anomalies.

### Disproportionality analysis

In order to evaluate the possible association between axitinib, lenvatinib, sorafenib, and sunitinib with AEs under gastrointestinal disorders, we used two methods for disproportionate analysis: ROR and PRR. ROR is mainly used to measure the imbalanced probability of reporting AEs for specific drugs compared with other drugs.

The calculation formula was:

$$ROR=\frac{a\times d}{b\times c}$$

(a) refers to the quantity of reports for particular drugs and particular AEs, (b) represents the quantity of reports for specific drugs and other AEs, (c) refers to the number of reports on other drugs and specific AEs, (d) represents the number of reports on other drugs and other AEs. PRR refers to the proportion of spontaneous reports of a specific drug associated with a specific adverse outcome divided by the corresponding proportion of other drugs. The calculation formula was:

$$PRR=\frac{a\times \left(c+d\right)}{c\times \left(a+b\right)}$$

Both ROR and PRR require that at least 5 cases (a ≥ 5) of particular drug and AEs to consider the calculated results valid. If ROR ≥2 and the lower limit of the 95% confidence interval (CI) ≥ 1, the signal is considered disproportionate, indicating that there may be a safety problem. These criteria ensure that the observed disproportion is not due to random variation (Montastruc et al., 2011). In our analysis, we systematically evaluate the ratio of ADRs reports of using four MTKIs in gastrointestinal disorders. The analysis results help to provide guidance for the correct use of drugs.

### Statistical analysis

A retrospective quantitative approach was adopted for this study. Descriptive analysis was conducted using Excel to evaluate the characteristics of individuals who experienced adverse reactions to the four MTKIs. The rate of ADR reporting for each MTKI was determined by dividing the number of ADR symptoms associated with that specific drug by the total number of ADR reports. The common ADRs linked to each medication were identified as those symptoms corresponding to the top 20 ADR report rates. The reported ADR symptoms for each drug were calculated, followed by a descriptive comparative analysis. Frequencies and percentages were utilized to classify the descriptive variables.

## Results

### Description of the studied cases

The initial documentation regarding adverse reactions to axitinib, lenvatinib, sorafenib, and sunitinib was first noted in the WHO-VigiAccess database during the years 2003, 2004, 2008, and 2013, respectively. By 2025, the WHO had gathered a cumulative total of 18,257, 28,819, 35,009, and 41,733 reports of ADRs linked to these four MTKIs, summing up to an overall total of 123,818 reports. Within these 123,818 ADR reports associated with the four MTKIs, as illustrated in Table 2, there were 7,052 cases in which the sex of the subjects was not specified. Importantly, the amount of ADR reports from males (73,485) significantly surpassed that from females (43,281), resulting in a male-to-female ratio of 1.70:1, highlighting a notable difference. When excluding reports that did not include age information, the age group most frequently reporting incidents was predominantly individuals aged 45–64 years. Additionally, most AEs were noted from the Americas, constituting 48.26% of the overall total. More information about the reporting years for each medication analyzed can be found in Table 2.

**TABLE 2:** Characteristics of ADR reports of four MTKIs.

Categories	Axitinib	Lenvatinib	Sorafenib	Sunitinib
Number of ADR reports	18,257	28,819	35,009	41,733
Female	4,955 (27.14%)	15,408 (53.46%)	9,297 (26.56%)	13,621 (32.64%)
Male	12,098 (66.26%)	12,703 (44.08%)	23,606 (67.43%)	25,078 (60.09%)
Unknown	1,204 (6.59%)	708 (2.46%)	2,106 (6.02%)	3,034 (7.27%)
0–27 days	3 (0.02%)	1 (0.01%)	13 (0.04%)	5 (0.01%)
28 days to 23 months	2 (0.01%)	1 (0.01%)	15 (0.04%)	1 (0.01%)
2–11 years	7 (0.04%)	13 (0.05%)	135 (0.39%)	19 (0.05%)
12–17 years	14 (0.08%)	44 (0.15%)	175 (0.50%)	58 (0.14%)
18–44 years	487 (2.67%)	800 (2.78%)	2,079 (5.94%)	1,790 (4.29%)
45–64 years	6,186 (33.88%)	7,488 (25.98%)	12,580 (35.93%)	13,359 (32.01%)
65–74 years	5,195 (28.45%)	7,137 (24.76%)	8,523 (24.35%)	10,544 (25.27%)
≥75 years	2,835 (15.53%)	4,125 (14.31%)	4,672 (13.35%)	5,190 (12.44%)
Unknown	3,528 (19.32%)	9,210 (31.96%)	6,817 (19.47%)	10,767 (25.80%)
Americas	11,689 (64.02%)	11,888 (41.25%)	12,420 (35.48%)	23,756 (56.92%)
Asia	3,313 (18.15%)	13,205 (45.82%)	14,021 (40.05%)	6,897 (16.53%)
Europe	3,135 (17.17%)	3,391 (11.77%)	8,239 (23.53%)	10,174 (24.38%)
Oceania	18 (0.10%)	295 (1.02%)	205 (0.59%)	357 (0.86%)
Africa	102 (0.56%)	40 (0.14%)	124 (0.35%)	549 (1.32%)
2025	195 (1.07%)	528 (1.83%)	127 (0.36%)	64 (0.15%)
2024	2,309 (12.65%)	7,706 (26.74%)	1,331 (3.80%)	984 (2.36%)
2023	1,922 (10.53%)	6,330 (21.96%)	2,019 (5.77%)	1,423 (3.41%)
2022	2,916 (15.97%)	4,468 (15.50%)	1,497 (4.28%)	2,472 (5.92%)
2021	2,276 (12.47%)	2,573 (8.93%)	1,399 (4.00%)	1,926 (4.62%)
2020	1,826 (10.00%)	2,169 (7.53%)	1,739 (4.97%)	2,046 (4.90%)
2019	993 (5.44%)	2,489 (8.64%)	2,053 (5.86%)	3,121 (7.48%)
2018	960 (5.26%)	1,228 (4.26%)	2,374 (6.78%)	3,785 (9.07%)
2017	692 (3.79%)	819 (2.84%)	4,033 (11.52%)	3,717 (8.91%)
2016	897 (4.91%)	423 (1.47%)	2,723 (7.78%)	3,272 (7.84%)
2015	1,486 (8.14%)	83 (0.29%)	3,333 (9.52%)	4,447 (10.66%)
before 2014	1,785 (9.78%)	3 (0.01%)	12,381 (35.37%)	14,476 (34.69%)

### Distribution of 20 SOCs of four MTKIs

Table 3 presents the reporting frequencies of 27 SOCs associated with four MTKIs. Axitinib exhibited elevated rates of adverse reactions in the categories of respiratory, thoracic, and mediastinal disorders, as well as in injury, poisoning, and procedural complications, when compared to the other three agents. Lenvatinib demonstrated higher adverse reaction rates in endocrine disorders, metabolic and nutritional disorders, renal and urinary disorders, and vascular disorders. Sorafenib showed increased rates of adverse reactions in hepatobiliary disorders and musculoskeletal and connective tissue disorders. Notably, the incidence of skin and subcutaneous tissue disorders was significantly higher for sorafenib than for the other agents. Sunitinib exhibited elevated adverse reaction rates across multiple SOC categories, including blood and lymphatic system disorders, gastrointestinal disorders, general disorders and administration site conditions, benign, malignant, and unspecified neoplasms (including cysts and polyps), nervous system disorders, and psychiatric disorders. Furthermore, the rates of ADRs exceeding 10% in the SOC were 11 for axitinib, 10 for lenvatinib, 10 for sorafenib, and 13 for sunitinib.

**TABLE 3:** ADR number and report rate of 27 SOCs of four MTKIs.

System organ classes	Axitinib (N = 18,257)	Lenvatinib (N = 28,819)	Sorafenib (N = 35,009)	Sunitinib (N = 41,733)
Blood and lymphatic system disorders	402 (2.20%)	1,265 (4.39%)	2,475 (7.07%)	5,785 (13.86%)
Cardiac disorders	859 (4.71%)	1,514 (5.25%)	1,612 (4.60%)	2,401 (5.75%)
Congenital, familial and genetic disorders	7 (0.04%)	9 (0.03%)	33 (0.09%)	38 (0.09%)
Ear and labyrinth disorders	176 (0.96%)	134 (0.46%)	298 (0.85%)	347 (0.83%)
Endocrine disorders	859 (4.71%)	2,187 (7.59%)	176 (0.50%)	1,584 (3.80%)
Eye disorders	382 (2.09%)	433 (1.50%)	573 (1.64%)	1,436 (3.44%)
Gastrointestinal disorders	8,560 (46.89%)	15,896 (55.16%)	21,436 (61.23%)	26,444 (63.36%)
General disorders and administration site conditions	8,982 (49.20%)	10,963 (38.04%)	15,701 (44.85%)	25,495 (61.09%)
Hepatobiliary disorders	675 (3.70%)	1,533 (5.32%)	2,311 (6.60%)	1,649 (3.95%)
Immune system disorders	157 (0.86%)	250 (0.87%)	289 (0.83%)	380 (0.91%)
Infections and infestations	1,293 (7.08%)	2,763 (9.59%)	3,169 (9.05%)	4,224 (10.12%)
Injury, poisoning and procedural complications	3,015 (16.51%)	2,660 (9.23%)	4,508 (12.88%)	4,901 (11.74%)
Investigations	3,825 (20.95%)	8,197 (28.44%)	7,164 (20.46%)	11,857 (28.41%)
Metabolism and nutrition disorders	1,985 (10.87%)	4,976 (17.27%)	4,688 (13.39%)	5,480 (13.13%)
Musculoskeletal and connective tissue disorders	2,180 (11.94%)	3,729 (12.94%)	4,954 (14.15%)	5,496 (13.17%)
Neoplasms benign, malignant and unspecified (incl cysts and polyps)	3,058 (16.75%)	3,052 (10.59%)	5,182 (14.80%)	8,206 (19.66%)
Nervous system disorders	3,391 (18.57%)	5,724 (19.86%)	5,651 (16.14%)	9,373 (22.46%)
Pregnancy, puerperium and perinatal conditions	2 (0.01%)	3 (0.01%)	6 (0.02%)	24 (0.06%)
Psychiatric disorders	858 (4.70%)	1,385 (4.81%)	1,936 (5.53%)	2,482 (5.95%)
Renal and urinary disorders	1,077 (5.90%)	2,294 (7.96%)	1,392 (3.98%)	3,028 (7.26%)
Reproductive system and breast disorders	123 (0.67%)	281 (0.98%)	476 (1.36%)	567 (1.36%)
Respiratory, thoracic and mediastinal disorders	3,220 (17.64%)	4,646 (16.12%)	4,960 (14.17%)	5,979 (14.33%)
Skin and subcutaneous tissue disorders	2,380 (13.04%)	3,459 (12.00%)	16,034 (45.80%)	9,798 (23.48%)
Social circumstances	73 (0.40%)	119 (0.41%)	198 (0.57%)	187 (0.45%)
Surgical and medical procedures	637 (3.49%)	512 (1.78%)	952 (2.72%)	492 (1.18%)
Vascular disorders	2,247 (12.31%)	4,151 (14.40%)	2,741 (7.83%)	4,543 (10.89%)
Product issues	44 (0.24%)	58 (0.20%)	79 (0.23%)	77 (0.18%)

### Disproportionality analysis based on gastrointestinal disorders

By observing and comparing the SOC distribution of four MTKIs, we found that these MTKIs exhibited the highest reported rates of adverse reactions under the categories of gastrointestinal disorders. To further compare these four medications, we conducted a disproportionate analysis using the ROR and PRR methods. Table 4 presents the results of this analysis, indicating the following ROR values for the four drugs: axitinib: 0.86 (0.84–0.89), lenvatinib: 1.04 (1.02–1.06), sorafenib: 1.08 (1.06–1.10), and sunitinib: 0.97 (0.96–0.99). Additionally, the PRR values for the four drugs were as follows: axitinib: 0.89 (0.87–0.91), lenvatinib: 1.03 (1.02–1.05), sorafenib: 1.06 (1.05–1.08), and sunitinib: 0.97 (0.98–0.99). These findings suggest that among the four MTKIs, axitinib had the lowest reported proportion of gastrointestinal disorders, whereas sorafenib exhibited a slightly higher reported proportion in comparison to the other drugs.

**TABLE 4:** Disproportionality analysis based on gastrointestinal disorders.

Drugs	ROR (95%CI)	PRR (95%CI)
Axitinib	0.86 (0.84–0.89)	0.89 (0.87–0.91)
Lenvatinib	1.04 (1.02–1.06)	1.03 (1.02–1.05)
Sorafenib	1.08 (1.06–1.10)	1.06 (1.05–1.08)
Sunitinib	0.97 (0.96–0.99)	0.97 (0.98–0.99)

### Most common ADRs of four MTKIs

Table 5 presents the 20 most frequently reported ADRs associated with the four MTKIs. The manifestations listed are preferred terms categorized within the SOC. Common ADRs for the four MTKIs include diarrhea, fatigue, death, hypertension, nausea, asthenia, weight loss, and vomiting. Among axitinib and sunitinib, death and disease progression are among the most frequently reported ADRs. Additionally, dysphonia and hypothyroidism warrant particular attention for both axitinib and lenvatinib. Furthermore, lenvatinib is associated with specific ADRs, including dehydration, arthralgia, and constipation. The reporting rates of rash and hepatocellular carcinoma for sorafenib are significantly higher than those for the other drugs. Sunitinib exhibits more pronounced hematological toxicity, primarily manifesting as thrombocytopenia, and may also lead to dysgeusia.

**TABLE 5:** Top 20 ADRs of four MTKIs.

Axitinib (N = 18,257)		Lenvatinib (N = 28,819)		Sorafenib (N = 35,009)		Sunitinib (N = 41,733)	
ADR	Report rate %	ADR	Report rate %	ADR	Report rate %	ADR	Report rate %
Diarrhoea	15.05%	Diarrhoea	12.02%	Diarrhoea	15.72%	Diarrhoea	11.89%
Fatigue	10.73%	Fatigue	10.34%	Rash	8.43%	Fatigue	10.48%
Death	9.00%	Hypertension	9.42%	Fatigue	7.83%	Nausea	7.65%
Hypertension	8.73%	Decreased appetite	8.20%	Decreased appetite	7.20%	Death	7.59%
Neoplasm progression	8.21%	Malignant neoplasm progression	7.35%	Nausea	6.40%	Neoplasm progression	7.52%
Off label use	6.48%	Nausea	7.10%	Off label use	6.27%	Disease progression	7.43%
Disease progression	6.04%	Blood pressure increased	6.34%	Asthenia	5.64%	Asthenia	6.20%
Nausea	5.70%	Vomiting	5.65%	Death	5.41%	Hypertension	6.04%
Dysphonia	5.58%	Asthenia	5.55%	Hepatocellular carcinoma	5.22%	Decreased appetite	5.61%
Decreased appetite	5.46%	Weight decreased	4.66%	Pain in extremity	4.67%	Vomiting	5.22%
Blood pressure increased	4.48%	Hypothyroidism	4.55%	Abdominal pain	4.58%	Stomatitis	4.10%
Asthenia	4.17%	Dehydration	3.97%	Alopecia	4.31%	Thrombocytopenia	3.99%
Weight decreased	4.06%	Headache	3.90%	Vomiting	4.30%	Dysgeusia	3.75%
Stomatitis	3.08%	Rash	3.08%	Pruritus	4.08%	Malaise	3.68%
Headache	3.05%	Stomatitis	2.89%	Weight decreased	4.04%	Weight decreased	3.41%
Vomiting	2.91%	Arthralgia	2.78%	Hypertension	3.95%	Platelet count decreased	3.19%
Hypothyroidism	2.56%	Constipation	2.58%	Blister	3.63%	Pain in extremity	3.10%
Malaise	2.55%	Dysphonia	2.53%	Pyrexia	3.26%	Blood pressure increased	3.04%
Rash	2.49%	Abdominal pain	2.49%	Skin exfoliation	2.87%	Anaemia	2.86%
Pain	2.46%	Death	2.38%	Pain	2.86%	Pain	2.79%

### Serious AEs of four MTKIs

**FIGURE 1:**
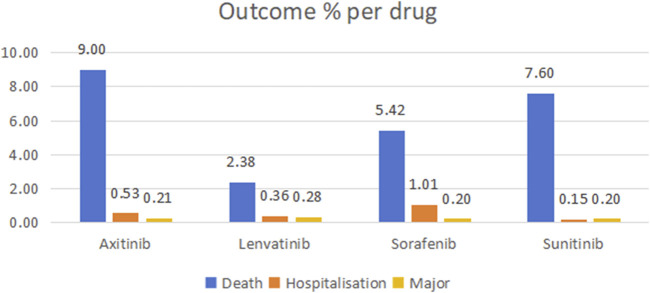
Outcomes for serious adverse events associated with four TIKs at the level of preferred terms (major events comprising life-threatening incidents, disabilities, and congenital anomalies).

Through WHO-VigiAccess, we can identify significant AEs associated with four MTKIs, including life-threatening occurrences, disabilities, and congenital malformations. The proportions of serious adverse reactions reported for axitinib, lenvatinib, sorafenib, and sunitinib were 9.74%, 3.02%, 6.63%, and 7.95%, respectively (Figure 1).

### The same and different points of common ADRs of four MTKIs

By examining the top 20 ADRs associated with each MTKI within the SOCs, a cumulative total of 163 identical signals was identified across the four MTKIs. All overlapping signals are detailed in Table 6. Gastrointestinal disorders and general disorders and administration site conditions emerged as the SOCs with the highest number of adverse signals, both ranking first in terms of frequency. For gastrointestinal disorders, the five most frequently reported reactions were flatulence, stomatitis, haematemesis, dry mouth, and oral pain. Meanwhile, for general disorders and administration site conditions, the top five reactions included condition aggravated, mucosal inflammation, pyrexia, disease progression, and oedema.

**TABLE 6:** Same ADRs between four MTKIs.

System organ classes	ADRS	Signal N
Blood and lymphatic system disorders	Anaemia, Thrombocytopenia	2
Cardiac disorders	Cardiac failure, Palpitations, Cardiac failure congestive, Atrial fibrillation, Cardiac disorder, Myocardial infarction	6
Endocrine disorders	Hypothyroidism	1
Eye disorders	Visual impairment, Vision blurred	2
Gastrointestinal disorders	Flatulence, Stomatitis, Haematemesis, Dry mouth, Oral pain, Mouth ulceration, Pancreatitis, Gastrointestinal haemorrhage, Abdominal pain, Haematochezia, Dyspepsia, Nausea, Abdominal distension, Diarrhoea, Constipation, Vomiting, Abdominal pain upper, Abdominal discomfort, Dysphagia, Glossodynia, Gastrointestinal disorder	21
General disorders and administration site conditions	Condition aggravated, Mucosal inflammation, Pyrexia, Disease progression, Oedema, Drug ineffective, Oedema peripheral, Feeling abnormal, Malaise, Gait inability, Chest pain, Chills, General physical health deterioration, Peripheral swelling, Fatigue, Gait disturbance, Drug intolerance, Swelling, Pain, Asthenia, Death	21
Hepatobiliary disorders	Liver disorder, Hepatic function abnormal	2
Immune system disorders	Hypersensitivity	1
Infections and infestations	Urinary tract infection, Pneumonia, Nasopharyngitis, Sepsis, Infection	5
Injury, poisoning and procedural complications	Fall, Product dose omission issue, Product use in unapproved indication, Contusion, Toxicity to various agents, Off label use, Product use issue	7
Investigations	Blood creatinine increased, Haemoglobin decreased, Weight increased, Heart rate increased, White blood cell count decreased, Weight decreased, Blood pressure increased, Alanine aminotransferase increased, Hepatic enzyme increased, Blood bilirubin increased, Aspartate aminotransferase increased, Blood glucose increased, Blood potassium decreased, Platelet count decreased	14
Metabolism and nutrition disorders	Feeding disorder, Dehydration, Hyperkalaemia, Decreased appetite, Hypophagia, Hyponatraemia	6
Musculoskeletal and connective tissue disorders	Back pain, Joint swelling, Myalgia, Arthralgia, Muscular weakness, Muscle spasms, Musculoskeletal pain, Bone pain, Pain in extremity	9
Skin and subcutaneous tissue disorders	Alopecia, Skin exfoliation, Dry skin, Pruritus, Blister, Erythema, Acne, Skin discolouration, Skin disorder, Rash, Skin ulcer, Urticaria, Hyperkeratosis	13
Neoplasms benign, malignant and unspecified (incl cysts and polyps)	Renal cell carcinoma, Renal cancer, Malignant neoplasm progression	3
Nervous system disorders	Balance disorder, Burning sensation, Memory impairment, Cerebral haemorrhage, Somnolence, Lethargy, Tremor, Headache, Dizziness, Seizure, Speech disorder, Neuropathy peripheral, Loss of consciousness, Ageusia, Cerebrovascular accident, Hypoaesthesia, Syncope, Paraesthesia, Dysgeusia	18
Psychiatric disorders	Insomnia, Anxiety, Confusional state, Eating disorder, Depression	5
Renal and urinary disorders	Proteinuria, Renal failure, Acute kidney injury, Renal impairment, Haematuria	10
Respiratory, thoracic and mediastinal disorders	Cough, Epistaxis, Haemoptysis, Pleural effusion, Dysphonia, Interstitial lung disease, Pulmonary oedema, Oropharyngeal pain, Pulmonary embolism, Dyspnoea	10
Social circumstances	Loss of personal independence in daily activities	1
Vascular disorders	Deep vein thrombosis, Haemorrhage, Hypertension, Thrombosis, Blood pressure fluctuation, Hypotension	6

Notably, when comparing the top 20 ADRs for each MTKI drug in the SOCs, each MTKI exhibited distinct PTs of ADRs in the following categories: general disorders and administration site conditions, investigations, and vascular disorders (Table 7). The number of unique symptoms reported for axitinib, lenvatinib, sorafenib, and sunitinib was 24, 25, 35, and 22, respectively.

**TABLE 7:** Different ADRs between four MTKIs.

System organ classes	Axitinib	Lenvatinib	Sorafenib	Sunitinib
Blood and lymphatic system disorders	Polycythaemia			Disseminated intravascular coagulation
Cardiac disorders		Cardiomyopathy	Tachycardia	Bradycardia, Pericardial effusion
Eye disorders				Lacrimation increased, Eye swelling, Eyelid oedema, Periorbital oedema
Gastrointestinal disorders	Tongue discomfort, Glossitis, Swollen tongue	Large intestine perforation, Intestinal obstruction, Intestinal perforation	Melaena, Pancreatitis acute, Faeces discoloured, Gastritis, Oesophageal varices haemorrhage	
General disorders and administration site conditions	Therapy partial responder	Decreased activity	Feeling hot, Unevaluable event, Adverse drug reaction	Multiple organ dysfunction syndrome, Face oedema
Hepatobiliary disorders	Hypertransaminasaemia, Hepatic cytolysis	Cholangitis, Gallbladder disorder, Cholecystitis acute	Hepatic cirrhosis, Hepatic pain	
Immune system disorders	Drug hypersensitivity			Decreased immune responsiveness
Infections and infestations	Candida infection	Cystitis, Septic shock	Gastroenteritis	
Injury, poisoning and procedural complications	Product dose omission in error, Wound, Intentional product use issue, Infusion related reaction, Product prescribing error	Incorrect dose administered		
Investigations	Laboratory test abnormal	Heart rate decreased, Blood magnesium decreased, Blood calcium decreased	Lipase increased, Blood alkaline phosphatase increased, Body temperature increased, Alpha 1 foetoprotein increased	Full blood count abnormal, Red blood cell count decreased, Full blood count decreased, Blood urea increased, Ejection fraction decreased
Metabolism and nutrition disorders		Tumour lysis syndrome, Electrolyte imbalance, Type 1 diabetes mellitus	Fluid intake reduced, Hypophosphataemia	
Musculoskeletal and connective tissue disorders		Fistula	Flank pain	Pain in jaw
Skin and subcutaneous tissue disorders	Night sweats		Dermatitis bullous, Rash papular, Palmoplantar keratoderma, Dermatitis, Rash erythematous, Drug eruption, Skin burning sensation	Hair colour changes, Yellow skin
Neoplasms benign, malignant and unspecified (incl cysts and polyps)		Cancer pain	Hepatocellular carcinoma, Metastases to bone, Acute myeloid leukaemia, Hepatic cancer, Thyroid cancer, Metastasis, Metastases to central nervous system, Metastases to lung	Gastrointestinal stromal tumour
Nervous system disorders	Cognitive disorder, Dysstasia, Movement disorder	Altered state of consciousness, Migraine		
Psychiatric disorders		Delirium, Hallucination		Mental status changes
Renal and urinary disorders				Chronic kidney disease
Reproductive system and breast disorders		Vaginal haemorrhage		
Respiratory, thoracic and mediastinal disorders	Throat irritation, Rhinorrhoea			
Surgical and medical procedures	Hospice care, Therapy interrupted, Nephrectomy			
Vascular disorders	Polycythaemia	Internal haemorrhage	Flushing	Pallor

## Discussion

In the treatment of RCC, axitinib, lenvatinib, sorafenib, and sunitinib have demonstrated significant clinical benefits. However, these MTKIs are also associated with a range of ADRs that can often be dose-limiting. By targeting not only the VEGFR but also other receptors such as the PDGFR and FGFR, these agents may induce hypertension, fatigue, gastrointestinal disturbances, and cardiovascular toxicities. Such ADRs can significantly affect patients’ quality of life, treatment adherence, and overall therapeutic outcomes (Shu et al., 2022; Zhang et al., 2024). Therefore, a systematic evaluation of their safety profiles is essential to optimize patient care and to address the ongoing challenge of rationally selecting the most appropriate MTKIs for RCC in clinical practice.

The Spontaneous Reporting System (SRS) serves an essential role in pharmacovigilance, facilitating the assessment of the safety of suspected AEs due to inherent limitations associated with clinical trials. Such limitations encompass stringent trial design, strict enrollment criteria, relatively small sample sizes, and short follow-up durations. Furthermore, data derived from clinical trials may not accurately represent real-world contexts, where variations in patient demographics and comorbidities can be significant. The SRS is crucial for detecting safety signals. Research related to the safety signals of numerous medications primarily relies on three major databases: the EudraVigilance Data Analysis System (EVDAS), the FDA Adverse Event Reporting System (FAERS), and WHO-VigiBase^®^ (Vogel et al., 2020). In 2015, the WHO launched WHO-VigiAccess, a platform that grants public access to the data compiled in VigiBase^®^, which is the WHO’s comprehensive repository of documented potential adverse effects linked to medicinal products. By analyzing information from the WHO-VigiAccess database, one can reveal previously unidentified connections between medications and AEs, as well as validate certain established clinical correlations (Yamoah et al., 2022). This research intends to assess the post-market AEs associated with four MTKIs using the WHO-VigiAccess database.

According to data from WHO-VigiAccess, 48.26% of AEs related to these four MTKIs were reported from the Americas, with only 815 report of AEs originating from Africa. Prior research has highlighted a significant issue with the low reporting rates of AEs in both Africa and Oceania (Alawadhi et al., 2012; Gidudu et al., 2020). The incidence of RCC is higher in regions with elevated income levels, likely due to improved access to medical resources and the increased prevalence of imaging diagnostics. In South Africa, the limited understanding of biopharmaceuticals among healthcare workers, coupled with high costs and complex procurement procedures, further exacerbates the barriers to the utilization of these medications (Hajjaj-Hassouni et al., 2012; Martelli et al., 2017; Kvamme et al., 2020). The African region has been noted for having the lowest incidence of reported AEs, which could be linked to insufficient social mobilization, restricted access to AE reporting mechanisms, and low levels of information system coverage. The number of ADR reports from men (73,485) significantly exceeded that of women (43,281), yielding a male-to-female ratio of 1.70:1. When excluding reports lacking information on age, the demographic groups with the highest rates of reported incidents were primarily those aged 45–64 years. This is consistent with epidemiological findings that RCC incidence is approximately twice as high in men compared to women, likely due to differences in sex hormones, gender-specific tumor microenvironments, and lifestyle factors. RCC is predominantly diagnosed in individuals aged 50–80 years (Capitanio et al., 2019). When analyzing the adverse reactions associated with these four MTKIs across different age groups, the lack of age data for 30,322 cases (24.49%) will inevitably impact the accuracy of our conclusions.

An AE reporting rate of ≥1% is typically regarded as common (Chen et al., 2019). The serious AEs associated with the four MTKIs—axitinib, lenvatinib, sorafenib, and sunitinib—include life-threatening events, disabilities, and congenital malformations. The mortality rates associated with these drugs are 9% for axitinib, 2.38% for lenvatinib, 5.42% for sorafenib, and 7.60% for sunitinib. Furthermore, sorafenib has a hospitalization rate of 1.01%. The most frequently reported ADRs for all four MTKIs include diarrhea, fatigue, death, hypertension, nausea, asthenia, weight loss, and vomiting. Notably, these four MTKIs exhibited the highest reported rates of adverse reactions within the gastrointestinal disorders category. An analysis of the ROR and PRR indicated that axitinib had the lowest reported proportion of gastrointestinal disorders, whereas sorafenib had a slightly higher reported proportion compared to the other drugs.

The adverse reaction most frequently encountered with the four MTKIs is diarrhea, which can significantly diminish treatment effectiveness and patient adherence, negatively impacting long-term outcomes for cancer patients, and in extreme cases, may even pose a threat to life (Keefe and Anthony, 2008). Diarrhea generally arises early in the treatment timeline, particularly during the initial month. The intensity of this condition is closely tied to the medication type and dosage. MTKIs can disrupt the blood supply to the intestinal lining by blocking the VEGFR, resulting in ischemia and hypoxia of the intestinal mucosa, which may trigger diarrhea. Additionally, patients on MTKIs therapy often develop submucosal fat accumulation in the gastrointestinal region, potentially linked to intestinal lymphangiectasia, which can exacerbate malabsorption and diarrhea (Liu et al., 2024). Managing diarrhea primarily depends on empirical symptomatic treatments, and educating patients is pivotal. It is vital for healthcare providers to discuss the possible side effects of MTKIs therapy with patients before starting treatment and to evaluate whether diarrhea is caused by MTKIs therapy during treatment. For those experiencing diarrhea, it is usually necessary to reduce or pause MTKIs therapy, and hospitalization may be considered if required. After diarrhea subsides, decisions regarding the resumption of treatment and dosage adjustments should be made based on the patient’s clinical situation. Regarding therapeutic options, probiotics and fecal microbiota transplantation might be utilized to adjust the gut microbiota and ease diarrhea (Ianiro et al., 2020). It is crucial to distinguish MTKI-induced diarrhea from infectious diarrhea and chemotherapy-related diarrhea, managing each case individually according to the severity and related complications (Benson et al., 2004).

MTKIs have the potential to cause hypertension in the management of RCC. This is likely due to the suppression of nitric oxide and prostacyclin synthesis that occurs when MTKIs inhibit VEGFR, resulting in the contraction of vascular smooth muscle. Moreover, another possible reason for hypertension associated with MTKIs is capillary rarefaction. This condition involves decreased vascular density, which heightens vascular resistance and, in turn, raises blood pressure (Hasinoff and Patel, 2010). It is essential for hypertensive patients, especially older adults with elevated baseline blood pressure, to establish effective blood pressure management before starting MTKI treatment. Patients who experience hypertension during therapy should follow standard protocols for hypertension management. Should blood pressure rise to alarmingly high levels, it is recommended to modify the MTKI dosage or pause the treatment. Studies suggest that the likelihood of hypertension may correlate with the dose of MTKIs, and hypertension itself could act as an important biomarker related to the effectiveness of the treatment (Ravaud and Sire, 2009). For instance, research utilizing real-world data from Japan demonstrated that patients with hypertension receiving MTKIs for RCC experienced enhanced overall survival (OS) and PFS over a 24-week period (Akaza et al., 2015). Furthermore, hypertension that develops during therapy is acknowledged as a standalone biomarker for the efficacy of sunitinib (Donskov et al., 2015). While axitinib and lenvatinib tend to show a greater frequency of hypertension compared to sunitinib, they typically pose a lower risk of cardiovascular issues. A thorough cardiovascular risk evaluation should be carried out before beginning MTKIs therapy, alongside consistent monitoring of blood pressure and potential cardiotoxic effects during the initial treatment phase (Bæk Møller et al., 2019).

Hypothyroidism might be linked to the suppression of the VEGFR, leading to the deterioration of capillary networks within the thyroid and a subsequent decrease in blood flow to thyroid cells as a result of the blockade of VEGFR (Liao et al., 2021). In addition, it can negatively influence thyroid function by lowering iodine absorption and inhibiting the activity of thyroid peroxidase. The onset of hypothyroidism usually takes time to manifest and may continue even after treatment has stopped (Wu and Huang, 2020). Consequently, during the early phases of treatment, it is advisable to closely monitor thyroid function and to inform patients about associated symptoms to allow for quick detection and treatment of thyroid-related issues. The clinical studies have verified that hypothyroidism among patients with RCC undergoing therapies like sunitinib or sorafenib acts as a favorable indicator of treatment success (Schmidinger et al., 2011; Baldazzi et al., 2012). Moreover, the emergence of skin rash is seen as a sign of increased effectiveness, with its side effects thought to relate to cross-activity among different kinases (Liu et al., 2013; Massey et al., 2015). Research has indicated that thoughtfully adjusting the medication dosage can substantially reduce adverse reactions while preserving therapeutic effectiveness. MTKIs hinder angiogenesis and disrupt the Wnt/β-catenin signaling pathway, affecting the differentiation of oral mucosal epithelial cells, preventing the regeneration of taste bud cells, and impairing nerve repair, which may result in conditions like stomatitiss, dry mouth, and dysgeusia (Boers-Doets et al., 2012; Epstein and Barasch, 2010; Naik et al., 2009). The inhibition of VEGF, recognized as a neurotrophic factor, could disrupt the conduction of taste nerves, while the broad inhibitory actions of these agents might further compromise olfactory capabilities (Carmeliet et al., 2013). Sunitinib has shown a notable association with dysgeusia, although the direct causal links remain ambiguous in current research.

These MTKIs are transported to the liver and metabolized by CYP3A4. So, avoid combining them with drugs that affect CYP3A4 activity. These drugs may alter the plasma concentrations of MTKIs, impacting efficacy or increasing toxicity (Bæk Møller et al., 2019; Wang et al., 2016). Also, a comprehensive, multidisciplinary approach can better manage MTKI - related drug ADRs, enhancing patients’' treatment experience and prognosis. Implementing regular multidisciplinary meetings to share patient information and discuss complex cases, utilizing a shared electronic health records system for real-time access to the latest patient data, jointly developing and carrying out patient education programs to ensure patients fully understand their treatment and potential ADRs, and collaborating on research projects to assess the effectiveness of management strategies and promote improvements in clinical practice.

The WHO-VigiAccess database, which operates on a voluntary basis for AE reporting, presents several challenges that hinder its ability to deliver a complete and thorough count of AEs. The database may not contain all the necessary information regarding reported incidents, which underscores the importance of enhancing the transparency of reporting practices. By improving the clarity and accessibility of the data provided to the public, stakeholders can engage in more effective screening for potential connections between pharmaceuticals and adverse reactions. This would also help prevent misguidance that could arise from incomplete or unclear information. The reliance on a spontaneous reporting system carries significant inherent limitations, primarily due to various biases that can affect the reporting process. These include notoriety bias, wherein more well-known drugs may receive disproportionate attention, selection bias, which can skew the data towards certain demographics, and under-reporting, which typically results in significant gaps in data collection (Faillie, 2019).

## Conclusion

Our research indicates that the adverse reaction reports submitted to the WHO and the FDA for these drugs highlight both common and specific adverse reactions. Clinicians must develop personalized treatment strategies that consider the adverse reactions associated with different drugs, as well as the unique circumstances of each patient, thereby encouraging the responsible use of these MTKIs.

## Data Availability

The original contributions presented in the study are included in the article/supplementary material, further inquiries can be directed to the corresponding authors.
